# Preferential targeting of cancer stem cells in the radiosensitizing effect of ABT-737 on HNSCC

**DOI:** 10.18632/oncotarget.7744

**Published:** 2016-02-26

**Authors:** Marion Gilormini, Céline Malesys, Emma Armandy, Patrick Manas, Jean-Baptiste Guy, Nicolas Magné, Claire Rodriguez-Lafrasse, Dominique Ardail

**Affiliations:** ^1^ Université Lyon I, Faculté de Médecine-Lyon-Sud, Oullins, France; ^2^ Laboratoire de Radiobiologie Cellulaire et Moléculaire, EMR3738, Oullins, France; ^3^ UMS3444 BioSciences Gerland-Lyon Sud, PBES, Lyon, France; ^4^ Hospices-Civils-de-Lyon, CHLS, Pierre-Bénite, France; ^5^ Institut de Cancérologie L. Neuwirth, St Etienne, France

**Keywords:** head and neck squamous cell carcinoma, ABT-737, radiation, Bcl-2 family, cancer stem cells

## Abstract

Head and neck squamous cell carcinomas (HNSCC) are common human malignancies with poor clinical outcomes. The 5-year survival rates for patients with advanced stage HNSCC have not changed appreciably in the past few decades, underscoring a dire need for improved therapeutic options. HNSCC is frequently characterized by overexpression of anti-apoptotic Bcl-2 family members. Increased levels of these anti-apoptotic proteins have been associated with radio- and chemoresistance and poor clinical outcome. The aim of this study was to evaluate combined effects of radiation and ABT-737, a BH3-mimetic molecule, in HNSCC. Although ABT-737, as a single agent, was largely ineffective at promoting HNSCC cell death, we found that combining ABT-737 and radiation induced strong synergistic apoptosis in HNSCC cell lines and delayed tumoral growth *in vivo*. Moreover, we demonstrated for the first time that ABT-737, alone or in combination with radiation, can efficiently eliminate cancer stem cells (CSCs). Altogether, our results indicate that therapy targeting anti-apoptotic Bcl-2 family members could be a highly effective potential adjuvant to radiotherapy capable of targeting CSCs in HNSCC and therefore overcoming cancer recurrence and metastasis.

## INTRODUCTION

Head and neck squamous cell carcinomas (HNSCC) are common human malignancies with poor clinical outcomes. The 5-year survival rates for patients with advanced stage HNSCC (TNM III-IV) have not changed appreciably in the past few decades, underscoring a dire need for improved therapeutic options. The major treatment options for HNSCC include surgery, radiation, and chemotherapy, depending on the stage and location of the tumor. Unfortunately, the aggressive treatment strategies necessitated for advanced stage HNSCC are associated with cytotoxicity and approximately 50% of patients who are initially treated will suffer recurrence within 2 years [1, 2] and expectation of cure is very limited. A key characteristic of advanced stage HNSCC is chemo- and radio-resistance resulting, in part, from aberrant overexpression of anti-apoptotic proteins of the Bcl-2 family which inhibit the intrinsic apoptosis pathway. A majority of HNSCC patients exhibit marked overexpression of Bcl-X_L_ [3] which has been shown to correlate with resistance to chemotherapy and radiotherapy and with poor clinical prognosis [4, 5]. In order to inhibit Bcl-X_L_ expression or function, antisense oligonucleotides [6] or short peptides (that bind to Bcl-X_L_ and Bcl-2 [7]) have been developed and shown to promote apoptosis signaling and cell death in HNSCC cell lines. More recently, the highly selective Bcl-X_L_/Bcl-2 inhibitor ABT-737 was shown to synergize with conventional chemotherapeutic drugs in killing HNSCC cells [8].

ABT-737 is a BH3-mimetic molecule that targets anti-apoptotic Bcl-2 family proteins (Bcl-2, Bcl-X_L_) thereby preventing them from binding the apoptosis activators (Bid, Bim, Puma) or sensitizers (Bad, Noxa). The displacement of the activator from Bcl-2 or Bcl-X_L_ by ABT-737 promotes Bax and Bak oligomerization and programmed cell death of malignant cells [9]. ABT-737 was shown to potently act as a single-agent drug on a number of cell lines [10–12] but also markedly increase the response to multiple chemotherapy agents *in vitro* [9, 10–15]. ABT-737 also showed good activity as a single agent in two small cell lung cancer xenograft models [9] and delayed morbidity in lymphoid as well as certain epithelial tumors *in vivo* [16–18]. Some studies have also shown that ABT-737 can enhance the radiosensitivity of solid resistant tumors [19, 20]. To our knowledge, there has been no prior study investigating the effect of ABT-737 in combination with radiotherapy for the treatment of HNSCC. Moreover, as cancer stem cells (CSCs) have been demonstrated to play a major role in local recurrence and metastatic spread in HNSCC [21], it appears that establishing innovative treatments targeting CSCs should be achieved in order to alleviate the morbidity and mortality of this pervasive disease. In the present study, we describe that ABT-737 combined with radiation synergistically induces apoptosis in HNSCC. We also describe the effects of ABT-737 on HNSCC stem cells and demonstrated a preferential cytotoxic activity towards these quiescent/slowly proliferating CSCs *in vitro* thus showing considerable promise to eradicate these therapy-resistant cells.

## RESULTS

### Sensitivity of HNSCC cell lines to ABT 737

**Figure 1:**
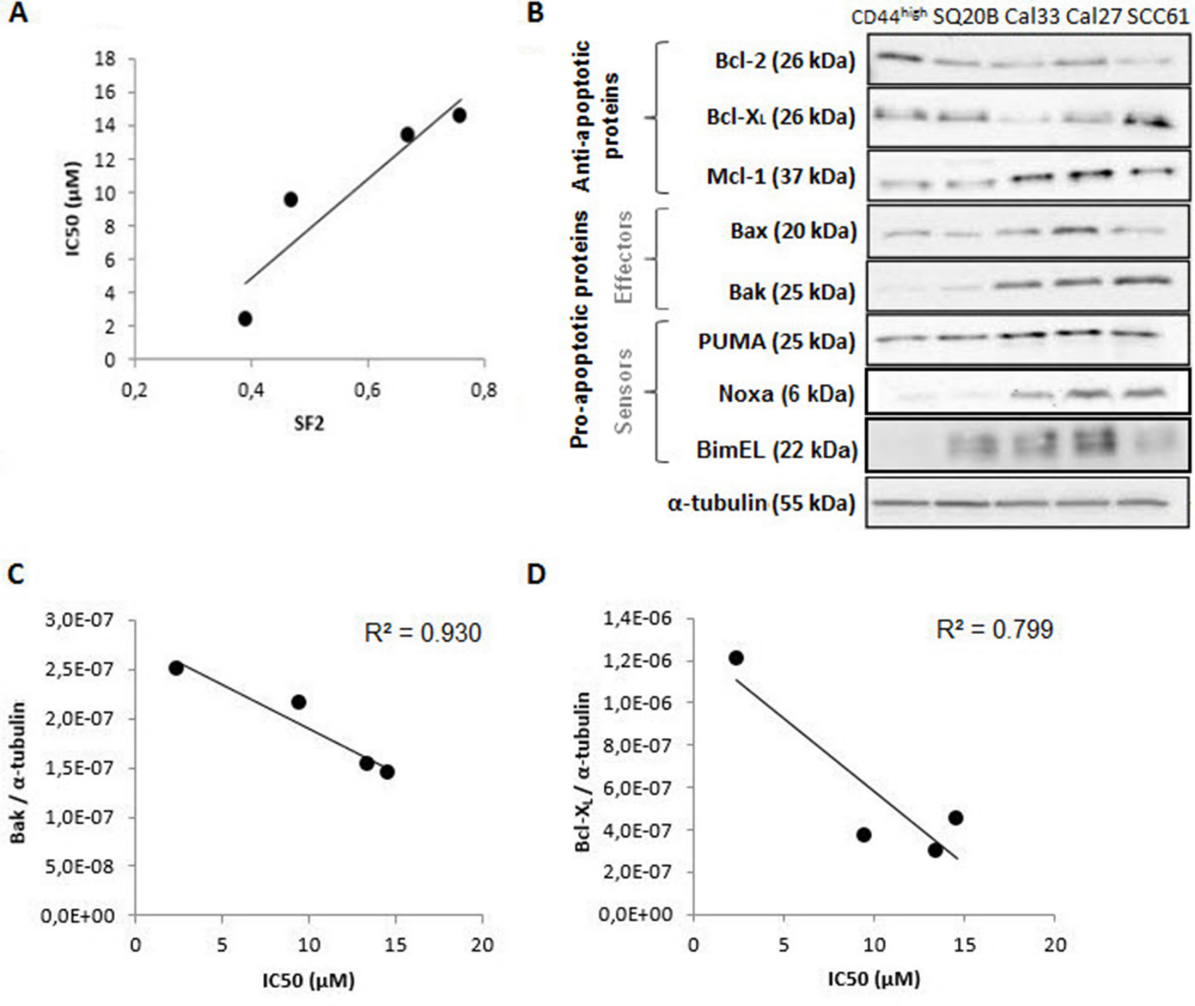
Study of the sensitivity of four human HNSCC cell lines to ABT-737 (**A**) Correlation between the sensitivity to ABT-737 (IC50) and the radiosensitivity of cell lines (SF2). (**B**) Expression of Bcl-2 family proteins of the four cell lines and the CSC sub-population (SP^+^/CD44^high^/ALDH^high^) was performed by Western blotting experiments. The correlation between sensitivity to ABT-737 (IC50) and (**C**) Bak expression, or (**D**) Bcl-X_L_ expression were normalized to the expression of α-tubulin.

We first determined 50% inhibitory concentrations (IC50) of ABT-737 in four HNSCC cell lines of graduate radiosensitivity (SF2 ranging from 0.39 to 0.76), defined as the dose of ABT-737 required to cause 50% loss in viability of cells at 48 h. All the cell lines had IC50 values ranging from 2 μM to 14 μM (Table 1). Moreover, a good correlation was obtained between the IC50 of ABT-737 and the SF2 of the four cell lines (R^2^ = 0.861) (Figure 1A). ABT-737 was previously shown to potently trigger cell death in certain tumoral cell types whereas other cells are less sensitive, a difference related to the differential expression of members of the Bcl-2 family. To check this, Western blot analysis (Figure 1B) showed that all the cell lines expressed Bcl-X_L_ and at a lesser extend Bcl-2, two primary targets of ABT-737. Concerning CD44^+^ cells (cancer stem-like cells), we can notice an overexpression of Bcl-2 (+100%) and Bcl-X_L_, at a lesser extend (+20%). In addition, they all express Mcl-1, a critical determinant for resistance to ABT-737, but at different levels. The sensitivity of our cell lines to ABT-737 suggests therefore that the Mcl-1 content is not high enough to inhibit ABT-737 effect. Considering the pro-apoptotic members of the Bcl-2 family, Bax is over-expressed when compared to Bak except for the SCC61 cell line. Interestingly, a good correlation was obtained between the Bak expression (R^2^ = 0.930) (Figure 1C) and between the Blc-X_L_ expression (R^2^ = 0.799) (Figure 1D) of the HNSCC cell lines studied.

**Table 1:** Characteristics of human head and neck squamous cell lines

Cell lines	SQ20B	Cal33	Cal27	SCC61
SF2	0.76	0.67	0.47	0.39
IC50 ABT-737 (μM)	14.56 (± 1.67)	9.48 (± 1.01)	13.44 (± 1.37)	2.38 (± 0.38)
Protein expression (relative blot density to α-tubuline) (.10^−7^)				
Bcl-2	1.11	4.28	1.89	3.94
Bcl-x_L_	4.52	2.98	3.71	1.21
Mcl-1	4.70	7.66	5.85	2.09
Bax	3.20	7.40	8.23	2.84
Bak	1.44	1.53	2.15	2.51
PUMA	0.70	0.89	0.40	0.98

### ABT 737 synergizes with irradiation to kill HNSCC cells

**Figure 2:**
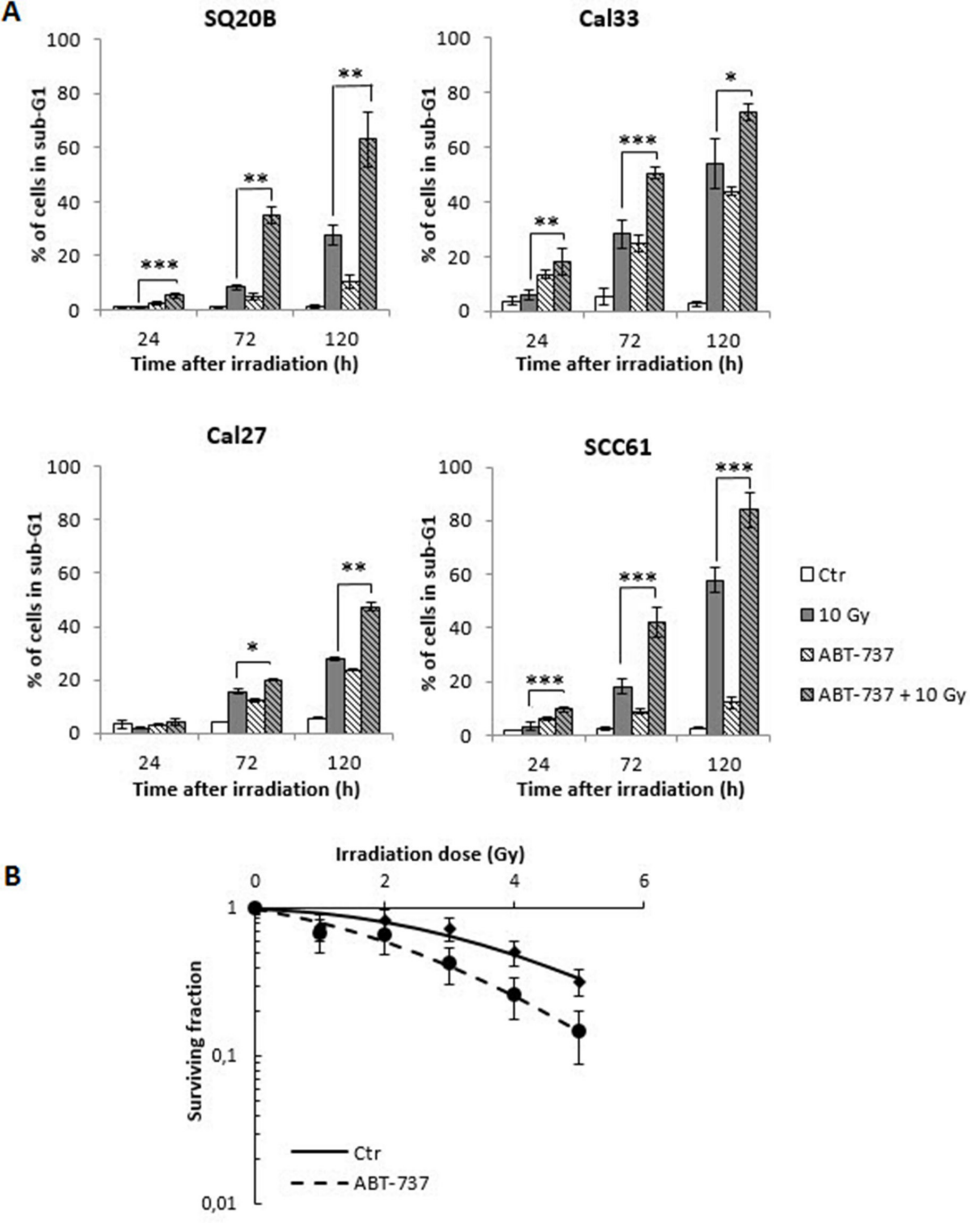
Radio-sensitization of HNSCC cell lines (**A**) Four HNSCC cell lines were treated with 0.1% DMSO (Control: Ctr) or 10 μM ABT-737 (ABT-737) 20 h after a 10 Gy irradiation. Cell death quantification was measured by the percentage of cells in the sub-G1 phase after 24 h, 72 h and 120 h after treatment +/– irradiation. (**B**) The clonogenic assay was performed on the most radioresistant cell line, SQ20B. The survival fraction at 2 Gy (SF2) was 0.81 (+/– 0.15) for control cells, and decrease to a value of 0.60 (+/– 0.16) after treatment of cells with 10 μM ABT-737. *p < 0.05; **p < 0.01.

In order to determine whether ABT-737 potentiates the effect of radiation therapy in HNSCC, the percentage of cells in the sub-G1 phase was quantified. As shown in Figure 2A, treatment of cells with ABT-737 alone led to a modest increase of sub-G1 cells ranging from 5% to 20% at 72 h, and 10% to 40% at 120 h, depending on the cell line. Although exposure to radiation alone led to a greater (SQ20B, SCC61) or equivalent (Cal27, Cal33) increase of the percentage of cells in the sub-G1 phase compared to ABT-737 alone, the combination ABT-737 with radiation resulted in a significant enhancement of hypodiploid cells, whatever the cell line considered. In order to confirm the radiosensitizing effect of ABT-737, a clonogenic assay was performed with the most radioresistant cell line, SQ20B, taken as a reference. As depicted in Figure 2B, a decrease of the SF2 value from 0.81 to 0.60 was obtained after treatment with ABT-737. Moreover, isobolographic analyses of cell survival show that ABT-737 and radiation effects are always synergistic, whatever the final level of cytotoxic efficiency (50 and 10% survival) and the applied dose of radiation ranging from 2 to 5 Gy (Supplementary Data S1). At the minimal dose of applied radiation (1 Gy), ABT-737 and radiation are additive at 50 and 10% survival efficiency.

### ABT-737 combined with irradiation activates apoptotic cell death

**Figure 3:**
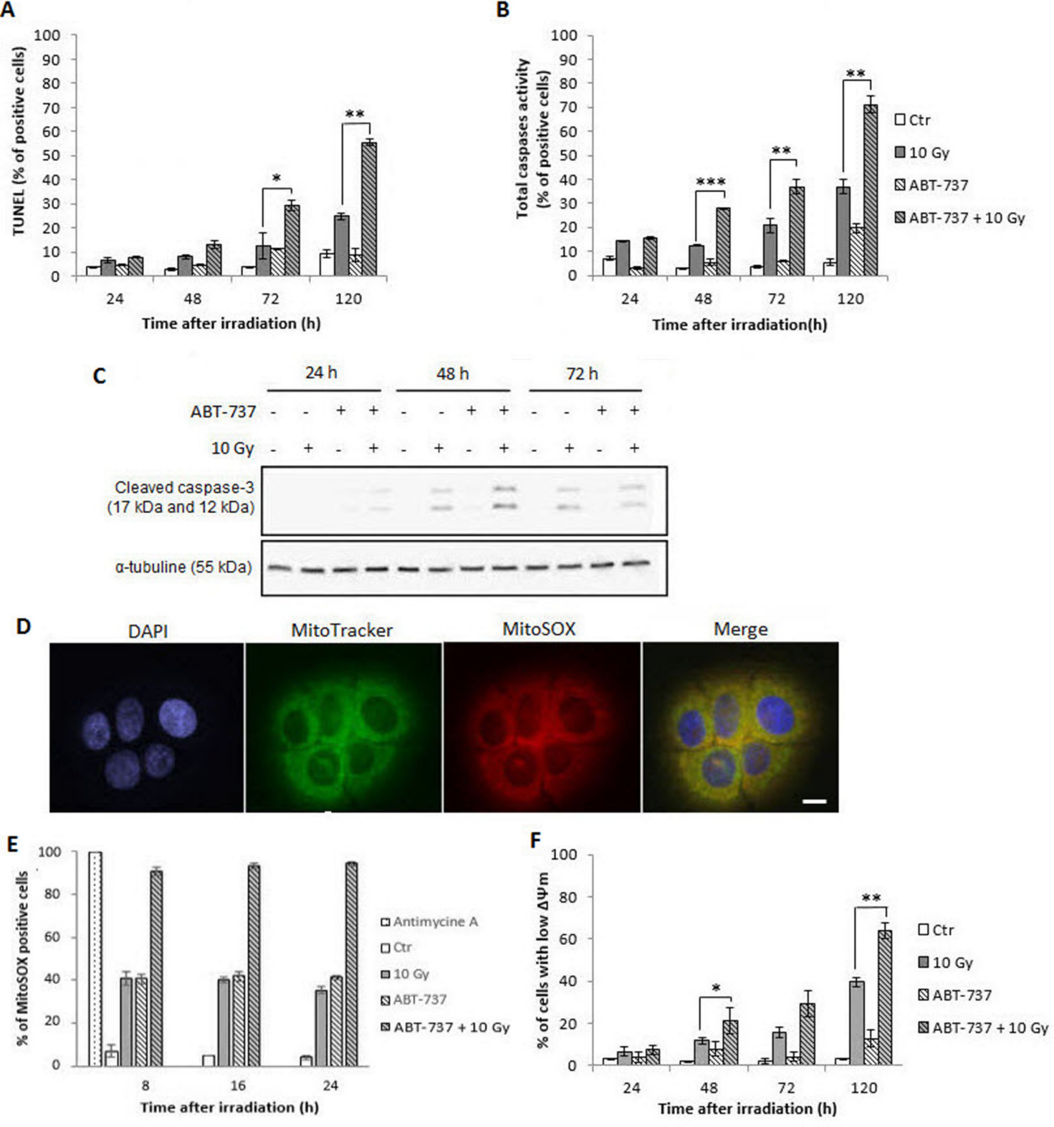
Treatment with ABT-737 before X-ray exposure triggers radiation-induced intrinsic apoptosis in SQ20B cell line and intra-mitochondrial oxidative stress SQ20B cells were treated with 0.1% DMSO (Control: Ctr) or 10 μM ABT-737, 20 h before a 10 Gy irradiation. After 24 h, 48 h, 72 h and 120 h, (**A**) cells were fixed and the percentage of TUNEL-positive cells were measured by flow cytometry analysis or (**B**) the percentage of cells having a caspase activity was measured on alive cells by flow cytometry analysis. (**C**) A Western blot analysis was performed to determine the specific activation of the procaspases-3 by cleavage. (**D**) The mitochondrial ROS production was validated with a positive (Antimycin A treated cells) control by fluorescence microscopy. Scale bar, 5 μm. (**E**) Specific mitochondrial ROS production was investigated by flow cytometry analysis using the MitoSOX labeling. (**F**) The loss of mitochondrial outer membrane potential (ΔΨm) was measured through a JC-1 staining on living cells. *p < 0.05; **p < 0.01; ***p < 0,001.

To confirm that the synergistic effect of ABT-737 and irradiation triggers apoptotic cell death, flow cytometry experiments were performed. Figure 3A shows that TUNEL-positive cells increased with time from 72 h after irradiation. Although no activation of apoptosis occurred with ABT-737 alone, a significant enhancement was obtained after the combined treatment (from 12 to 30% of positive cells at 72 h and 25 to 58% at 120 h). Similar results were obtained with total caspases activity measurement (Figure 3B) and specific activation of caspase-3 (Figure 3C). All these results confirmed those obtained after the analysis of the sub-G1 peak described above.

Given the fact that mitochondria represent the preferential cellular location of Bcl-2 and Bcl-X_L,_ two targets of ABT-737, and the hub of apoptosis-related events [22], we examined if ABT-737 combined with irradiation could trigger an intra-mitochondrial oxidative stress. The specific dye MitoSOX that can penetrate live cells was used to visualize (Figure 3D) and quantitate (Figure 3E) the production of superoxide anions by mitochondria [23]. After checking the specificity of the MitoSOX labeling with antimycin A, flow-cytometry analysis show that ABT-737 resulted in a significant and sustained increase of MitoSOX fluorescence from 8 h after treatment and that radiation strongly reinforced the effect of ABT-737. Additional flow-cytometric analysis of JC-1-stained SQ20B cells (Figure 3F) revealed that the combined treatment led to the loss of mitochondrial transmembrane potential in a time-dependent manner, from 48 h after treatment. All these results demonstrate that the combination ABT-737-irradiation radiosensitize SQ20B cells through triggering of the intrinsic apoptotic pathway.

### ABT-737 combined with irradiation alters expression levels of Bcl-2 family members

**Figure 4:**
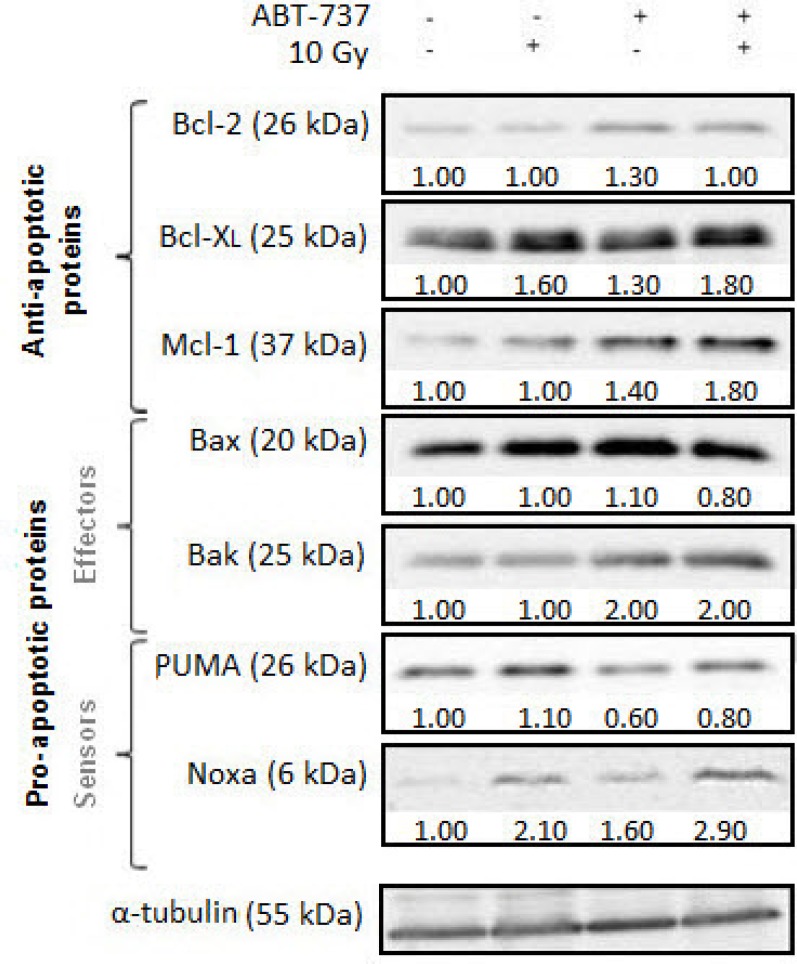
ABT-737 combined with irradiation alters expression levels of Bcl-2 family members Representative Western blot analysis of Bcl-2 family members were performed 24 h after irradiation. Tubulin is shown as a loading control. Blots are representative of three independent experiments.

We next investigated the synergistic effect of ABT-737/radiation on the expression levels of Bcl-2 family members (Figure 4). Treatment with ABT-737 alone did not substantially alter the level of the pro-apoptotic protein Bax but did cause a modest increase of the anti-apoptotic proteins Bcl-2, Bcl-X_L_ and Mcl-1, more importantly with respect to the protein Noxa. By contrast, a striking up-regulation of Bak was obtained with ABT-737 alone. Only Bcl-X_L_ and Noxa protein levels exhibited changes in response to radiation alone. Treatment combining ABT-737 with radiation resulted in consistent up-regulation of Bcl-X_L_ and Mcl-1 compared with ABT-737 alone. Regarding Noxa, radiation strongly enhanced (+105%) its expression relative to control cells while treatment with ABT-737 alone resulted in a more modest increase (+59%). A cumulative effect on Noxa expression was obtained (+190%) after the combined treatment. Thus, these data suggest that radiation induces an overexpression of Noxa which could inactivate Mcl-1 and make them sensitive to ABT-737.

### ABT-737 dramatically increases cell death in the cancer stem cells sub-population

**Figure 5:**
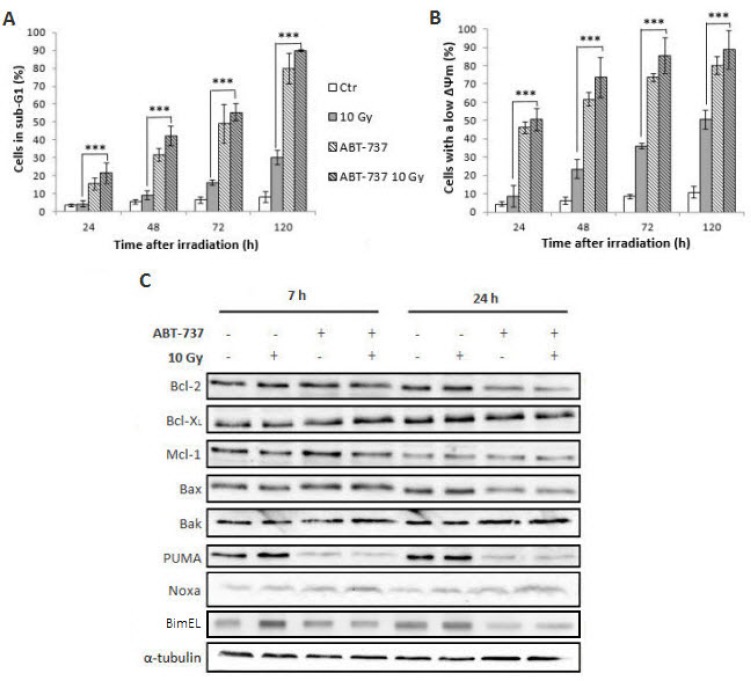
Treatment with ABT-737 increases X-ray-induced apoptosis of the SP+/CD44^high^/ALDH^high^ cells sub-population A SP^+^/CD44^high^/ALDH^high^ sub-population sorted from the SQ20B cell line was treated with DMSO 0.1% (Control: Ctr) or 10 μM ABT-737 20 h before a 10 Gy irradiation. 24 h, 48 h, 72 h and 120 h after irradiation, (**A**) the percentage of sub-G1 cells and (**B**) the loss of mitochondrial outer membrane potential (ΔΨm) were measured by flow cytometry analysis. (**C**) The expression of Bcl-2 family proteins in the SP^+^/CD44^high^/ALDH^high^ sub-population was performed by Western blot analysis, 7 hours and 24 hours after irradiation, respectively. Tubulin is shown as a loading control. Blots are representative of three independent experiments ***p < 0.001.

In order to overcome radioresistance and potential relapse of HNSCC, we next investigated the efficiency of ABT-737 combined with photon exposure on a sub-population of cells (SP^+^/CD44^high^/ALDH^high^) isolated from the parental SQ20B cell line whose cancer stem cells properties have been described in details elsewhere [24]. As depicted in Figure 5A, the treatment of SP^+^/CD44^high^/ALDH^high^ cells with ABT-737 alone resulted in an increase of the percentage of cells in the sub-G1 phase with respect to time (from 15% at 24 h up to 79% at 120 h). Surprisingly, only a very small additional increase was obtained when ABT-737 was combined with radiation therapy (15 to 19% at 24 h and 79 to 90% at 120 h). If these results are compared with those obtained in the parental SQ20B cell line, we can notice the high efficiency of ABT-737 against SP^+^/CD44^high^/ALDH^high^ cells (79% of the cells in the sub-G1 phase compared to 10% obtained in the parental cell line). Next, the involvement of mitochondria in the triggering of SP^+^/CD44^high^/ALDH^high^ cell death was checked by the measurement of Δψm loss. Figure 5B shows that in more than 47% of these cells, mitochondria are affected at 24 h up to 75% at 120 h. As reported, no significant additional increase is obtained when ABT-737 is used in combination with radiation. These results are very promising because they demonstrate that ABT-737 is a potent cell death inducer, particularly effective against HNSCC cancer stem cells. By comparison, while 63% of parental SQ20B cell line died after ABT-737 and irradiation, 89% of SP^+^/CD44^high^/ALDH^high^ cell death is obtained under the same experimental conditions. SP^+^/CD44^high^/ALDH^high^ cells are thus more susceptible to ABT-737-induced apoptosis than the parental cell line. Considering the expression levels of Bcl-2 family members in SP^+^/CD44^high^/ALDH^high^ cells (Figure 5C), a small increased expression of all the members (except Bak and PUMA) of the Bcl-2 family is observed at 7 h but only Noxa is considerably increased even after treatment with ABT-737 alone (180%).

### ABT-737 combined with ionizing radiation delays the tumoral growth *in vivo*

**Figure 6:**
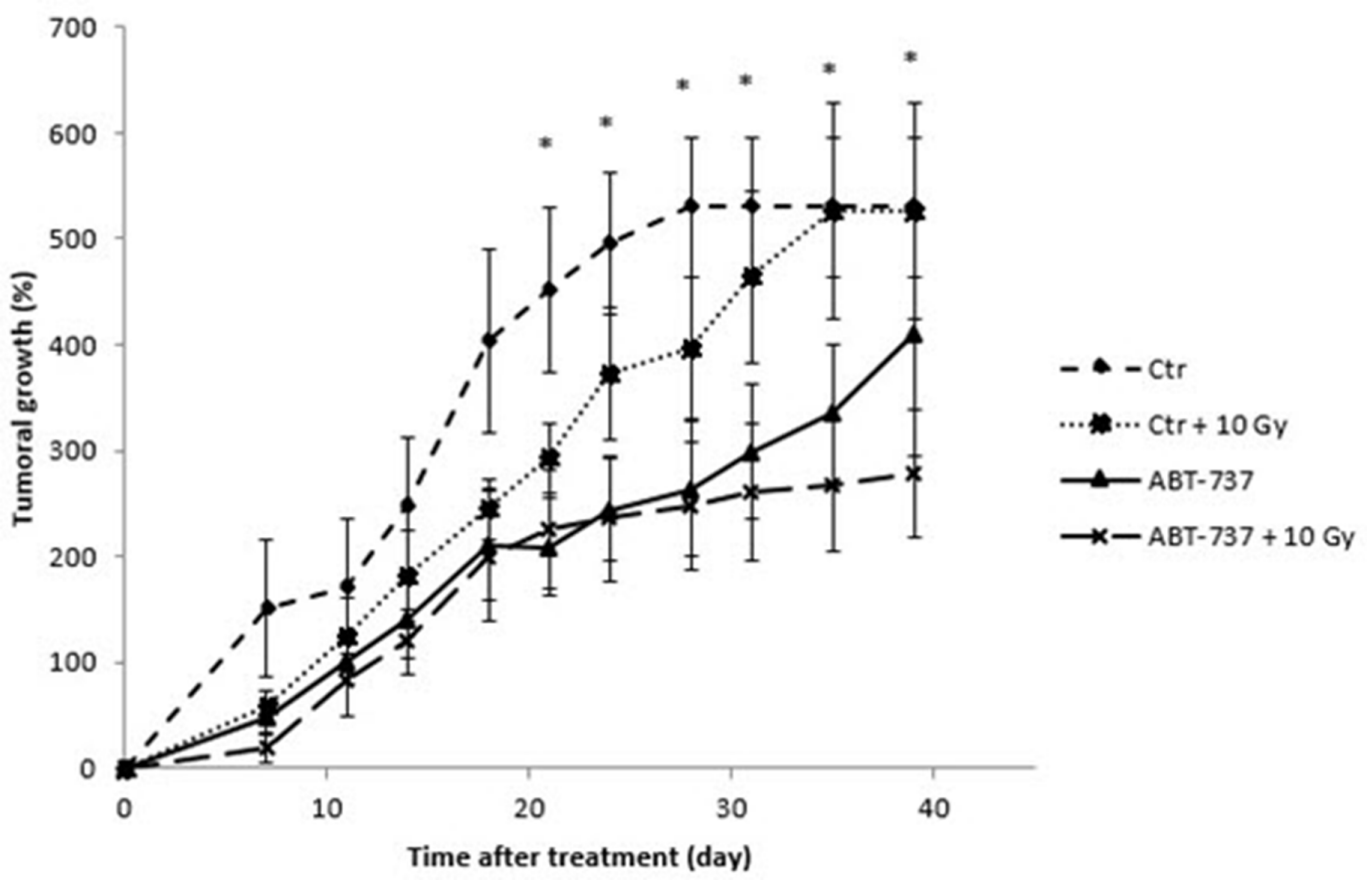
Treatment with ABT-737 delays tumoral growth *in vivo* Mice were treated intraperitoneally with 0.2% DMSO (Control: Ctr) or with ABT-737 (20 mg/kg/day) 1 hour before irradiation of tumor xenografts (2 Gy/day; Ctr + 10 Gy and ABT-737 + 10 Gy) and this during 5 consecutive days. Tumor volume was monitored up to 40 days. *p < 0.05.

To investigate whether ABT-737 could reverse acquired radioresistance of HNSCC *in vivo*, we used a heterotopic xenograft tumor model in nude mice. As shown in Figure 6, the tumors treated with ionizing radiation grew at a slower rate than those of the control group just after the week of treatment, but re-grew rapidly a few weeks after. Interestingly, the treatment with ABT-737 alone seems to have a more pronounced delayed effect. Finally, the tumors in the ABT-737 plus radiation group grew like the ABT-737 group until 30 days, and more slowly thereafter. Taken together, these results suggest that ABT-737 has a similar effect as irradiation on SQ20B xenograft tumor growth whereas the combination ABT737 + irradiation is much more effective than ABT-737 or radiation alone.

## DISCUSSION

Developing effective strategies to circumvent HNSCC resistance and to improve clinical outcome has proven to be a challenging task. In this report, we show the synergistic effect of ABT-737, an inhibitor of anti-apoptotic Bcl-2 proteins, with radiation therapy which results in the radio-sensitization of HNSCC cell lines, including CSCs *in vitro*, and *in vivo* in a HNSCC xenograft model. This study also demonstrate that ABT7-37, alone or in combination with radiation therapy, showed a preferential cytotoxicity towards HNSCC CSCs, which is a promising approach to reduce cancer relapse and metastasis.

ABT-737 binds Bcl-2 and Bcl-X_L_ with high affinity and has shown single-agent efficacy against multiple cell lines as well as in combination with standard therapy in human leukemia and multiple myeloma models [3, 25], lymphoma [26], melanoma [13], cholangiocarcinoma [14] or HNSCC [8]. Except some reports on Hela cells [27], non-small-cell-lung cancer cells [19] and breast cancer cells [20, 28], no more data are available on a synergistic effect of BH3-mimetics with radiation, especially on HNSCC. Therefore, we first evaluated ABT-737 efficacy in a panel of four HNSCC cell lines of graduate radio-sensitivity and observed a correspondingly range of sensitivity to ABT-737 *in vitro*. All the cell lines used in this report expressed Bcl-2, Bcl-X_L_ and Mcl-1 at different levels. Although no significant correlation between the expression of each anti-apoptotic protein of the Bcl-2 family and their sensitivity to ABT-737 and/or individual cell line radio-sensitivity could be identified, a positive correlation between their individual radio-sensitivity and their sensitivity to ABT-737 was obtained. It is now well documented that there is no single determinant of cell sensitivity to ABT-737 [29] and the expression of Bcl-2 or Bcl-X_L_ is a necessary but not a sufficient condition to confer drug sensitivity [30]. As an example, Mcl-1 confers resistance to ABT-737 because of the poor affinity of this drug for Mcl-1. Silencing Mcl-1 by RNA interference increases the sensitivity to ABT-737 [31, 32] while ectopic expression of Mcl-1 can render cells resistant to ABT-737 [33]. As emphasized by our results, expression of Mcl-1 in our cell lines is not sufficient to cause resistance to ABT-737 thus suggesting that complex interactions rather than simply the expression pattern of Bcl-2 proteins plays a role in determining the sensitivity of Mcl-1-expressing cells to ABT-737 [34]. Furthermore, exposure of SQ20B cells to ABT-737, alone or in combination with radiation, was in contrast found to up-regulate Mcl-1 expression, a result that was previously reported in lymphoma and breast cancer cells [35, 36].

Interestingly, a good correlation was found between pro-apoptotic Bak expression and radio-sensitivity in the four HNSCC cell lines but not between Bak and Bax expression. Although the significance of this finding should be explored in the future, a prognostic value of Bak expression in oral tongue squamous cell carcinoma had already been reported [37].

Despite the fact that the resistance, either to chemotherapy and radiotherapy, of HNSCC cells is due in part to the expression of anti-apoptotic members of the Bcl-2 protein family, including Bcl-X_L_ and Bcl-2 at a lesser extent, the sensitivity of HNSCC to ABT-737, in synergy with cisplatin and etoposide, has only been reported in one work [8]. As no data are available about using ABT-737 with radiation to date, we demonstrate for the first time, here, the high efficiency of this synergistic combination in HNSCC. Taking the most radio-resistant cell line (SQ20B) as a reference, we also show that the combination of ABT-737 with radiation markedly enhanced the sensitivity to radiation through the induction of intrinsic apoptotic cell death. Although ABT-737 alone was unable to significantly trigger apoptosis, radiation causes apoptosis occurrence, assessed by total caspases activity and DNA fragmentation, but remained relatively low. Used in combination, ABT-737 and radiation work synergistically, as assessed by dramatic increase in mitochondrial membrane depolarization, caspase-3 activation and DNA fragmentation.

Our studies also reveal that Noxa expression was markedly up-regulated after treatment with ABT-737 plus radiotherapy. Similar up-regulation of Noxa by ABT-737 plus chemotherapy has been already observed in melanoma cells [13], colorectal cancer cells [38], ovarian carcinoma cells [39] and H196 SCLC cells that are highly resistant to ABT-737 alone [18], thus conferring sensitivity of the cells to this drug.

It is noteworthy that Noxa is known to bind with high affinity to Mcl-1, but not Bcl-X_L_ or Bcl-2 [40]. This suggests that in our cellular model, Noxa up-regulation should functionally inactivate Mcl-1, causing displacement of pro-apoptotic proteins, such as Bak, bound to the Mcl-1 protein and promoting synergism by this combination. Moreover, it was recently demonstrated [41] a direct interaction between Noxa and either Bax or Bak during apoptosis induction thus supporting the role of Noxa as a potential activator of both Bax and Bak. Noxa has also been previously suggested [42] to be activated in response to γ-radiation in a p53-dependent as well as –independent manner. Thus, in HNSCC cells, repression of cellular Mcl-1 via Noxa up-regulation by ABT-737 alone or radiation alone may explain their highly potent synergism in promoting, for example, a Bak-dependent cell death.

Poor survival rates and treatment failures are common features in the management of advanced stage diseases of HNSCC. The initiation, growth, recurrence, and metastasis of HNSCC and other cancers have recently been related to the presence of CSCs, a subpopulation which displays the ability to undergo self-renewal and differentiation and hence have the ability to initiate tumorigenesis and support ongoing tumor growth [43]. Therefore, targeted elimination of CSCs has been considered a new conceptual framework for head and neck cancer treatment [44, 45]. Although it has been proposed that targeting self-renewal pathways in CSCs, such as the Wnt, Notch, and Hedgehog pathways [46], or specific CSC markers, such as CD133 (Prominin-1) [47], CXCR1 [48, 49], and CD44 [50], may offer therapeutic benefits to cancer therapy, evidence for the benefits of blocking these pathways and markers in HNSCC has not been reported so far. However, recent data offer a preclinical proof-of-concept for targeting the c-Met/FZD8 signalling axis as a CSC-directed therapy to improve HNSCC treatment [51]. Furthermore, inhibition of Rac 1 expression and signalling was shown to interrupt metastatic process due to anoikis restoration and decrease of cell migration [52]. Several studies have also focused on manipulating the apoptotic machinery to eradicate tumour-initiating cells, either by intervening in the extrinsic or intrinsic pathways [53] through the use of XIAP inhibitors [54] or down-regulation of cFLIP [55]. In this paper, we demonstrated for the first time that ABT-737, alone or in combination with radiation, exhibited a powerful and preferential cytotoxic activity towards HNSCC stem cells. This can be explained by the simple observation of Bcl-2 overexpression in CSCs (see Figure 1). Our data could therefore argue for a Bcl-2-dependent anti-apoptotic threshold in HNSCC CSCs. In accordance with our data, a preferential elimination of CSCs after Bcl-2 family proteins-targeted strategies was recently demonstrated in other models such as leukemia stem cells [56, 57] and non-small cell lung cancer [58].

Although it was demonstrated that anti-apoptotic proteins of the Bcl-2 family display regulatory functions on migration as well as invasiveness of colorectal cancer cells [59], it occurred independently of their anti-apoptotic effects. Future studies are therefore needed to explain how ABT-737 can both trigger HNSCC stem cells death and to impair their migration and invasion.

Finally, owing to its ability to preferentially kill HNSCC stem cells, ABT-737 treatment, in association with radiation, may not result in a fast macroscopic reduction of tumour size but may have long-term beneficial effects on the rates of tumour growth and relapse. Clinical trials designed to specifically evaluate this endpoint will help to clarify the usefulness of inhibition of Bcl-2 family pro-apoptotic members in the treatment of HNSCC.

## CONCLUSION

Based on the data of our study, we conclude that the combination of ABT-737 with radiation triggers the caspase-dependent mitochondrial apoptosis in HNSCC. Moreover, owing to its ability to preferentially kill HNSCC stem cells, this combined therapy is expected to significantly circumvent HNSCC resistance and reduce cancer relapse and metastasis.

## MATERIALS AND METHODS

### Cell culture

Four human-derived HNSCC cell lines (SQ20B, SCC61, Cal27 and Cal33) were used and cultured in a 5% CO_2_ atmosphere. SQ20B and SCC61 were obtained from John Little Laboratory (Harvard School of Public Health, Boston, USA); Cal27 and Cal 33 were obtained from G. Milano (Dept of Oncopharmacology, Centre A. Lacassagne, Nice, France). The SQ20B cell line is derived from a larynx epidermoid carcinoma; the SCC61, Cal27 and Cal33 cell lines are derived from tongue squamous carcinomas. SQ20B and SCC61 cell lines were cultured in Dulbecco's modified Eagle's medium containing 4.5 g/L of glucose supplemented by 10% of fetal calf serum (FCS), 0.04 mg/l of hydrocortisone, 100 U/ml of penicillin and 0.1 g/L of streptomycin. Cal27 and Cal33 were cultured into the same medium but without hydrocortisone.

### Cancer stem cells isolation

### Flow cytometry sorting for Hoechst efflux

The protocol used was previously described by Bertrand *et al*. [24]. Briefly, the SQ20B cell line is trypsinized and incubated with Hoechst 33342 dye (Sigma-Aldrich) at 25 μg/l during 90 min at 37°C. Cells are then washed with PBS and re-suspended at a concentration of 10^7^ cells/ml in PBS. Hoechst efflux, distinctive of side population (SP) cells, is measured with a FACS-LSRII (BD Biosciences, excitation: 355 nm, emission: blue: 450 nm, red: 675 nm). Simultaneously, a negative control is prepared under the same experimental conditions by adding 50 mM Verapamil, an inhibitor of ABC carriers. This control must show a disappearance of the SP cluster. After sorting, SP cells are transferred in a flask and maintained at 37°C in a 5% CO_2_ atmosphere.

### Expression of CD44 and ALDH by flow cytometry

SP cells are labeled with a CD44 antibody and ALDH detection kit (Aldefluor^™^ kit, StemCell, 01700) in order to select the CD44^high^/ALDH^high^ sub-population. Briefly, cells are trypsinized, washed with PBS and re-suspended into Aldefluor buffer. Cells are then incubated with Aldefluor reagent 45 minutes at 37°C. After a wash, cells are incubated for 10 min at 4°C with an anti-CD44-APC mouse monoclonal antibody (1/1 000, Miltenyi Biotech), washed in PBS and sorted with a FACS-LSRII flow cytometer (BD Biosciences). Thereafter, sorted cells are cultured at 37°C in a 5% CO_2_ atmosphere.

### ABT-737 treatment

ABT-737 (Abbott Laboratories, Illinois) was dissolved into Dimethylsulfoxide (DMSO) at a concentration of 10 mM. Cells were seeded and treated 8 hours after. Control cells were treated in parallel with 0.1% of DMSO.

### Irradiation procedures

Cell monolayers were irradiated in a X-rad 320 irradiator (PXI, North Brandford, CT) at a dose rate of 2 Gy/min, an energy of 250 kV and an intensity of 15 mA. Cell irradiations were realized 20 hours after the treatment by ABT-737 or DMSO.

### Cell growth measurement

72 hours after irradiation, cells were fixed with 96% ethanol and colored with a Cristal Violet solution (0.1% in 20 mM HEPES, pH 6). Next, colored cells were lysed with a 1% SDS solution and the absorbance was measured at 560 nm.

### Western blot analysis

Cell monolayers were trypsinized and lysed with a cold lysis buffer (150 mM NaCl, 50 mM Tris-HCl (pH 8.0), 1% Triton X-100) containing protease and phosphatase inhibitors (Roche). After 30 min incubation on ice, lysates were centrifuged at 15,000 g for 20 min. The total protein concentration in the lysates was measured using the bicinchoninic acid (BCA) assay (Sigma-Aldrich). The antibodies used were: anti-caspase-3 (Cell Signalling, 9662), anti-Bcl-2 (Santa Cruz, sc-509), anti-Bcl-X_L_ (Cell Signaling, 2762), anti-Mcl-1 (Santa Cruz, sc-819), anti-Bax (Santa Cruz, sc-493), anti-Bak (Cell Signaling, 3814), anti-PUMA (Cell Signaling, 4976), anti-Noxa (Enzo Life Sciences, ALX-804-408), anti-α-tubulin (Santa Cruz, sc-8035) and anti-GAPDH (Biodesign, H-86-504M). Equal protein loading was confirmed with α-tubulin or GAPDH expression. Relative protein quantification was done with Image Gauge Fujifilm software (Fuji).

### Analysis of clonogenic cell survival

Cell lines were seeded in 25 cm^2^ flasks at different cell densities depending on the dose of radiation applied. Cells were treated with 10 μM ABT-737 for 7 hours after seeding and irradiated 13 hours after treatment at doses varying from 0 to 5 Gy. Cell survival was assessed by the standard colony formation assay as described by Beuve *et al*. [60].

### Cell cycle analysis

Cells were fixed with 70% ethanol and 4′,6′-diamidino-2-phenylindole (DAPI) staining was used at 1 μg/mL to analyze the cell cycle distribution using a FACScan flow cytometer (BD LSRII flow cytometer, BD Biosciences).

### TUNEL assay

Apoptosis was assayed with the DeadEnd^™^ Fluorometric TUNEL System (Promega, G3250). Briefly, cells were fixed with 1% paraformaldehyde in PBS for 20 minutes on ice and then permeabilized with 70% ethanol for 4 hours minimum at −20°C. Cells were then incubated with the reaction mixture for 1 hour at 37°C. After washing with PBS, cells were stained with 5 μg/ml PI containing 0.5 mg/ml DNase-free RNase A for 30 minutes at room temperature and analyzed using the FACScan flow cytometer at an excitation wavelength of 520 nm and an emission wavelength of 600 nm.

### Activated caspases assay

CaspACE^™^ FITC-VAD-FMK *In situ* Marker (Promega, G7462) was used to quantify activated caspases. Following trypsination, the inhibitor is added and incubated for 20 minutes at room temperature. After washing with PBS, cells are resuspended in PBS and analyzed using the FACScan flow cytometer at an excitation wavelength of 488 nm and an emission wavelength of 530 nm.

### Mitochondrial membrane potential measurement

The mitochondrial membrane potential (ΔΨm) was measured with the JC-1 (5,5′,6,6′-tetrachloro-1,1′,3,3′-tetraethylbenzimidazolylcarbocyanine iodide) dye (Sigma-Aldrich, T4069). After trypsinization of the cells, the JC-1 dye is added at a concentration of 5 μg/ml and incubated for 20 minutes at 37°C. Then, cells are analyzed using the FACScan flow cytometer at an excitation wavelength of 488 nm and an emission wavelength of 525 nm.

### Total reactive oxygen species formation assay

Total Reactive Oxygen Species (ROS) were measured through dihydroethydium (DHE, Sigma-Aldrich, D7008) oxidation. After trypsinization of the cells, 4 μM DHE is added in PBS and incubated for 10 minutes at 37°C. The reaction is stopped by transferring tubes on ice. Then, cells are analyzed using the FACScan flow cytometer at an excitation wavelength of 520 nm and an emission wavelength of 600 nm.

### MitoSOX^™^ red (mitochondrial superoxide indicator) assay

Before treatment with Antimycin A, cells are incubated with 50 nM MitoTracker^®^ Green FM (Invitrogen, M7514) diluted into Hank's Balance Salt Solution (HBSS) buffer containing calcium and magnesium (Gibco) for 30 min at 37°C. Cells are then washed with HBSS buffer and treated with 10 μM Antimycin A (Sigma-Aldrich, A8674-25MG). After washing in HBSS buffer, cells are treated with 5 μM MitoSOX (Molecular Probes, Carlsbad, CA, M36008) for 30 min at 37°C. Then cells are washed with HBSS buffer and fixed with 4% paraformaldehyde (PFA) for 20 min at room temperature and are stained with 1 mg/l DAPI for 10 min at room temperature. Labeled cells are visualized by fluorescence microscopy (Axio Imager Z2, Zeiss). Alternatively, results can also be analyzed by flow cytometry after staining for 30 min with 5 μM MitoSOX before analysis. Thereafter, cells are trypsinized and fluorescence intensity is measured by flow cytometry at an excitation wavelength of 488 nm and an emission wavelength of 575 nm (LSR III, BD Biosciences).

### *In vivo* study

The *in vivo* study protocol (number 00746.02) was accepted by the ethics committee of the Claude Bernard Lyon 1 University, the CECCAPP. We used 6-week-old females NODE-SCID mice (Charles River Laboratory, L'Arbresle, France). 3 × 10^6^ SQ20B cells were injected subcutaneously in the intern face of the left leg of the mice under isoflurane anesthesia. 3 weeks after the xenograft, mice were randomized (see Supplementary Data S2) into 4 groups in order to be treated as follows: the control group (*n* = 11), the ABT-737 group (*n* = 11), the 10 Gy group (*n* = 8) and the ABT-737 + 10 Gy group (*n* = 7). The control group was treated by 2% DMSO in physiological serum injected intra-peritoneally. The ABT-737 group was treated as the same, with 20 mg/kg ABT-737 diluted in physiological serum. The 10 Gy group was first treated with 2% DMSO in physiological serum injected intraperitoneally, and the tumor was irradiated at 2 Gy by day. Finally, the ABT-737 + 10 Gy group was treated with 20 mg/kg ABT-737 in physiological serum, injected intraperitoneally, and the tumor was irradiated at 2 Gy by day. These treatments were realized every day during 5 consecutive days. During the week of treatment, mice were weighted every day and twice a week after treatment (mice were sacrificed if 10% of the starting weight was lost) together with the size and aspect of the tumor (mice were sacrificed if the tumoral volume exceeded 1,200 cm^3^ or if the tumor was necrotic).

### Statistical analysis

Dose-response interactions between radiation and ABT-737 were evaluated using the classical isobolographic method described by Steel and Peckham [61]. The theoretical basis and procedure of the isobologram method have been described in detail [62]. The coordinates of the experimental point are the ABT-737 concentration and the radiation dose which, when combined, give the level of efficacy. For a given level of efficacy (% survival) an “envelope of additivity” curve was calculated from the dose effect curves of ABT-737 combined to irradiation (5 doses) and from the dose-effect curves of radiation alone (5 doses). If the experimental point falls above, beyond or under the limits of the “envelope of additivity”, ABT-737 and radiation combination give rise to antagonistic (Ant), additive (+) or synergistic (Syn) effects, respectively.

Each experiment was realized in triplicates. Statistical analysis was realized with the Student's *t* test. Significant results have a *p* value < 0.05 (*), < 0.01 (**) or < 0.001 (***).

## SUPPLEMENTARY MATERIALS TABLE AND FIGURE


