# Supplementary material for: Preferential targeting of cancer stem cells in the radiosensitizing effect of ABT-737 on HNSCC
Source: Oncotarget. 2016 Feb 26;7(13):16731–44. doi: 10.18632/oncotarget.7744 (PMC4941347; doi:10.18632/oncotarget.7744)
Supplement: Supplementary file 1 [file oncotarget-07-16731-s001.pdf]

Preferential targeting of cancer stem cells in the radiosensitizing effect of ABT-737 on HNSCC

Supplementary Materials

Supplementary Data S1: Isobolographic analyze on the SQ20B cell line

Irradiation dose (Gy)	Isobolographic analyze	
	Survival 10%	Survival 50%
1	Additive	Additive
2	Synergistic	Synergistic
3	Synergistic	Synergistic
4	Synergistic	Synergistic
5	Synergistic	Synergistic

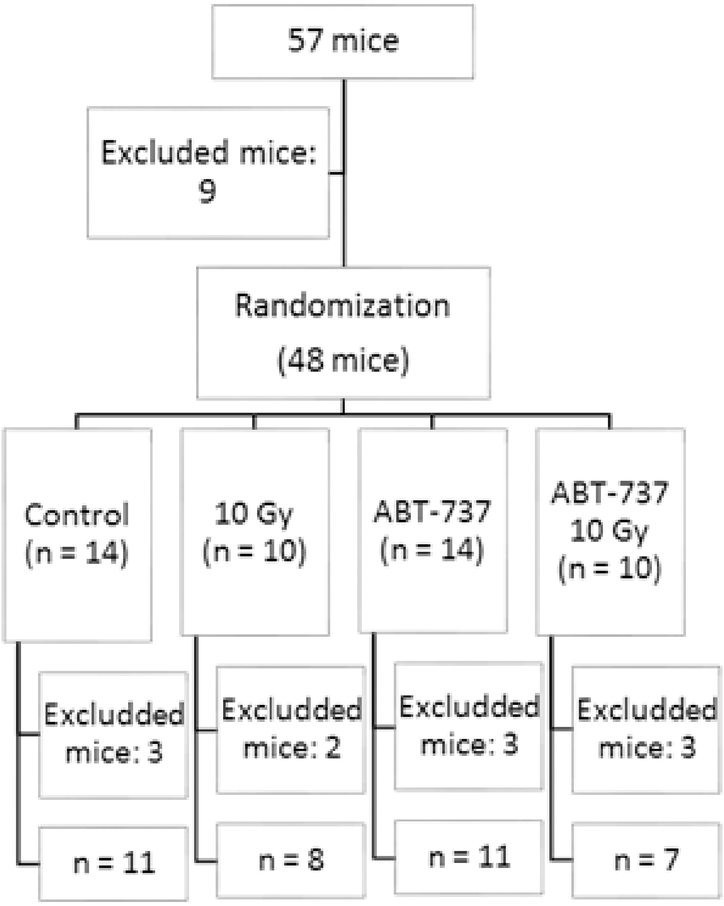

Supplementary Data S2: Randomization diagram. Before the randomization, 9 mice were excluded because of their low tumoral volume (< 60 cm^3). The randomization and treatments were realized on 48 mice. After treatment, some mice were excluded for different reasons (important edema that modified the tumoral volume measurement, necrosis of tumor, tumoral volume > 400 cm^3 at the first day after treatment).
